# NGS transcriptomic analysis uncovers the possible resistance mechanisms of olive to *Spilocea oleagina* leaf spot infection

**DOI:** 10.3389/fpls.2023.1219580

**Published:** 2023-07-17

**Authors:** Annalisa Marchese, Bipin Balan, Daniela Antonina Trippa, Floriana Bonanno, Tiziano Caruso, Valeria Imperiale, Francesco Paolo Marra, Antonio Giovino

**Affiliations:** ^1^ Department of Agricultural, Food and Forest Sciences, University of Palermo, Palermo, Italy; ^2^ Research Centre for Plant Protection and Certification, Council for Agricultural Research and Economics, Palermo, Italy; ^3^ Department of Architecture (DARCH), University of Palermo, Palermo, Italy

**Keywords:** *Olea europaea*, *Peacock’s eye infection*, molecular resistance mechanism, abiotic and biotic resistance, RNA-sequencing, candidate genes, breeding *Spilocea oleagina* resistant olive genotypes

## Abstract

*Spilocea oleagina* is a dangerous obligate fungal pathogen of olive, feared in the Mediterranean countries, causing *Peacock’s eye* or *leaf spot infection*, which can lead to a serious yield loss of approximately 20% or higher depending on climatic conditions. Coping with this disease is much more problematic for organic farms. To date, knowledge on the genetic control of possible mechanisms of resistance/low susceptibility is quite limited. In this work, comparative transcriptomic analysis (RNA-seq) was conducted in leaf tissues of a low susceptible cultivar Koroneiki and a high susceptible cultivar Nocellara del Belice, both tested in the field using the NaOH test, considering two stages—”zero sign of disease” and “evident sign of infection”. Cultivars showed a very large number of differentially expressed genes (DEGs) in both stages. ‘Koroneiki’ showed an extensive hormonal crosstalk, involving Abscisic acid (ABA) and ethylene synergistically acting with Jasmonate, with early signaling of the disease and remarkable defense responses against *Spilocea* through the over-expression of many resistance gene analogs or pathogenesis-related (PR) genes: non-specific lipid-transfer genes (nsLTPs), LRR receptor-like serine/threonine-protein kinase genes, GDSL esterase lipase, defensin Ec-AMP-D2-like, pathogenesis-related leaf protein 6-like, Thaumatin-like gene, Mildew resistance Locus O (MLO) gene, glycine-rich protein (GRP), MADS-box genes, STH-21-like, endochitinases, glucan endo-1,3-beta-glucosidases, and finally, many proteinases. Numerous genes involved in cell wall biogenesis, remodeling, and cell wall-based defense, including lignin synthesis, were also upregulated in the resistant cultivar, indicating the possible role of wall composition in disease resistance. It was remarkable that many transcription factors (TS), some of which involved in Induced Systemic Resistance (ISR), as well as some also involved in abiotic stress response, were found to be uniquely expressed in ‘Koroneiki’, while ‘Nocellara del Belice’ was lacking an effective system of defense, expressing genes that overlap with wounding responses, and, to a minor extent, genes related to phenylpropanoid and terpenoid pathways. Only a Thaumatin-like gene was found in both cultivars showing a similar expression. In this work, the genetic factors and mechanism underlying the putative resistance trait against this fungal pathogen were unraveled for the first time and possible target genes for breeding resistant olive genotypes were found.

## 1 Introduction

The olive tree (*Olea europaea* L.), a millenary plant typical of the Mediterranean area, is appreciated for its fruits and oil. It is considered a very rustic species and is generally resistant to abiotic and biotic adversities. However, it can be attacked by various fungal and bacterial pathogens in favorable climatic conditions, which can cause significant production losses. The sanitary defense of the olive grove is an important objective for the economy of the crop as any damage to the plants reduces the vegetative activity and can compromise the fruits, with negative effects on the quality of the finished product and the production costs. Among the fungal pathogens of the olive tree, *Spilocaea oleagina* (Castagne) Hughes (recently named *Venturia oleaginea* (Castagne), Rossman et al., 2015) is much feared for the damage it causes in the Mediterranean area (Mekuria et al., 2001; Obanor et al., 2005, Gonzalez-Dominguez et al., 2017, Buonaurio et al., 2023) because this disease, called *Peacock’s eye or leaf spot infection*, can lead to serious yield loss of approximately 20%. The disease is particularly harsh in densely planted orchards of susceptible olive cultivars and in nurseries (Graniti, 1993; Schena et al., 2011).

The life cycle of the pathogen *Spilocaea oleaginea* depends on climatic conditions such as temperature and humidity (Obanor et al., 2005; Obanor et al., 2008; Rhimini et al., 2020, Buonaurio et al., 2023). *Peacock’s eye* is generally associated with a high humidity rate typically occurring during the winter period. During the summer, the disease is not present due to warmer temperatures that prevent germination of the spores of the fungus (Obanor et al., 2011; Rhimini et al., 2020). The growth of the fungus was found to be prevalent in the period from late autumn to spring and less so in the period from the beginning of July to mid-November (Viruega and Trapero, 1999). *Peacock’s eye* is usually more abundant in the lower parts of the olive trees (Graniti, 1993) and grows on the surface of the leaves; in susceptible cultivars it also severely attacks the fruits (Miller, 1949). The fungus spreads through mobile zoospores. Once in contact with the host cuticle, the conidia germinate and emit a pro-mycelium that perforates it, thus invading the plant tissue into sub-cuticular spaces (sub-cuticular parasites) with the production of a hyphal system. This location is ideal for *Spilocaea oleaginea* as it derives nourishment necessary for its growth from cell wall degradation, in particular waxes, cutin, lipids, cellulose, and pectin. In addition, the mycelium exploits the presence of the cuticle for protection against dehydration and radiation. The lesions form dark brown round spots (2–15 mm in diameter) that become necrotic and surrounded by concentric yellowish or light brown halos (Graniti, 1993, Buonaurio et al., 2023). These spots, particularly those on the leaf pages, are velvety when the fungus fructifies. On the fruit, when attacked, the fungus manifests itself with brownish spots several millimeters in diameter (Miller, 1949; Lanza et al., 2017). Fruit infection affects ripening, negatively influences oil yield, and causes detrimental injuries to table olives (Salman et al., 2014; Lanza et al., 2017, Buonaurio et al., 2023). Many studies have investigated the role of fungal cutinases in the invasion of plant tissues by enzymatically degrading the cuticle (e. g. Martin and Juniper, 1970; Matsuda et al., 1998).

Currently, only the application of synthetic fungicides (containing copper) allows disease control in the field throughout olive-growing regions of the world (e.g., Teviotdale et al., 1989; Shabi et al., 1994; Obanor et al., 2008; Sistani et al., 2009; Salman et al., 2014). However, the application of chemicals is not desirable in relation to human and environmental health factors. Because the accumulation of pesticides based on copper in the soil causes toxicity in the soil microbiome, in EU countries, the use of copper in olive groves was limited in January 2019 to no more than 28 kg/ha in seven years, at an average of 4 kg/ha/year (EU rule 1981/2018). Therefore, finding a source of genetic resistance represents the only effective way to stop *Spilocea oleagina* disease (Anton and Laborda, 1989).

Some olive cultivars exhibit low susceptibility to *Spilocea oleagina* and show an increase in the content of polyphenols and oleuropein derivatives, hydroxytyrosol, rutin, hydroxycinnamic acids, and flavonols having a fungitoxic effect (Mekuria et al., 2001; Báidez et al., 2007; Rahioui et al., 2009; Aabidine et al., 2010); however, the defense mechanisms involved are not well understood. Benitez et al. (2005), screening a resistant cultivar and a susceptible cultivar using the different display method (DD), demonstrated that olive resistance to *S. oleagina* is genotype-dependent and that an active defense response involving the accumulation of hydrogen peroxide (H_2_O_2_) leads to the synthesis of Salicylic acid and other hormones showing complex overlaps with wound responsive gene pathway.

A recent histochemical investigation on the possible involvement of a hypersensitive response (HR) conducted by Lanza et al. (2017) demonstrated that olive shows a weak plant defense reaction, limited to the local activation of polyphenol oxidase (PPO)-catalyzed phenolic oxidation in just a few upper epidermal cells, with no HR. Lanza et al. (2017) also speculated that *S. oleagina* may have evolved a system that enables it to evade the plant defense system due to its subcuticular localization and probably due to a molecular mechanism of simultaneous degradation and uptake of cutin monomers that avoid detection by plant receptors. In this way, the fungus can persist and proliferate within the thin layer of the cuticle without triggering a massive defense response by the host. However, deeper analysis at the molecular level is needed to verify this hypothesis (Lanza et al., 2017).

Nowadays, analysis of the transcriptome using next-generation sequencing (NGS) techniques allows accurate and extensive identification of resistance/tolerance genes and potential biomarkers (Giovino et al., 2015). Scientific research is actively seeking to develop diagnostic methods that make it possible to identify the disease early in the field, before the onset of symptoms (Martinelli et al., 2019), through the analysis of gene expression and the identification of biomarkers in the very early stages of infection. The early-stage identification of the disease could allow prompt intervention to limit the diffusion of the fungal agent; for the reasons given above, it is extremely important to study this further.

This research aimed to discover the possible resistance mechanisms of olive against *Spilocea oleagina* and to discover genes of resistance and putative biomarkers linked to the infection by analyzing a low susceptible cultivar, Koroneiki (Buonaurio et al., 2023), and a highly susceptible cultivar, ‘Nocellara del Belice,’ using next-generation sequencing approaches.

## 2 Materials and methods

### 2.1 Evaluation of S. oleagina disease incidence in a field collection and cultivar selection

From 2020 to 2021, the evaluation of *S*. *oleagina* infection on olive cultivars grown in an experimental collection field (P. Messina, Sciacca, AG, Italy, partner of the SPREMO project) was carried out, evaluating the infection at several months: September, December, March, April, and May.

It was evident that, usually at the end of summer (September), trees did not show signs of disease, whereas in April there was a disease outbreak. The presence of *S. oleagina* infection was determined according to the method described by Abuamsha et al. (2013). A total of 100 leaves were selected in April and September from each tree by randomly picking them from the four sides of the central portion of the canopy (1.5 meters above the ground). Leaves with visible and invisible symptoms were dipped in 5% NaOH for 2 min at 50–60° C (Shabi et al., 1994; Hernández et al., 1998). Disease incidence was evaluated by determining the percentage of infected leaves (0 to 100) following the method of Abuamsha et al. (2013). According to the percentage of infected leaves, genotypes can be divided into 4 groups: a) low susceptible—showing less than 30% infected leaves; b) medium susceptible—with a range of 30%–60% infected leaves; c) medium–highly susceptible—with a range of 61%–90% infected leaves, and d) highly susceptible with over 90% infected leaves. In the collection field during the two years of observation, the cultivar Koroneiki showed a putative resistance to *Spilocea oleagina* (less than 20% infected leaves), whereas the main Sicilian table olive cultivar Nocellara del Belice (Barone et al., 2014) was particularly susceptible (more than 90% infected leaves). For this reason, these highly divergent cultivars in terms of susceptibility were chosen for the RNA-seq analysis.

In September, leaves from both cultivars were symptom-free, as determined using the NaOH test.

### 2.2 Total RNA extraction

Leaf samples were taken in the field from two eight-year-old trees—one being the resistant cultivar Koroneiki (KT) and the other the susceptible cultivar Nocellara del Belice (NB)—and immediately stored in liquid nitrogen at two stages: “no signs of disease” (stage 0, indicated as T1), collected on the 24th of September and “evident presence of disease” (stage 3, indicated as T3), collected on the 21st of April (**Figure S1**). Subsequently, the plant material (100 mg for each sample) was pulverized in liquid nitrogen using a pestle and mortar. A Spectrum Plant Total RNA (Sigma) Kit was used to extract the total RNA. The total RNA was quantified and its purity was evaluated using a Nanodrop’ (ND1000 Thermo Fisher Scientific). Overall, the yield and quality were good, and approximately 200 ng/μL was obtained.

### 2.3 cDNA reverse transcription for Real Time

An iScript ™ gDNA BioRad cDNA synthesis kit was used for RNA retro-transcription (RT) following the recommended protocol. The reaction was performed in a final volume of 20 μL containing 4 μL of buffer, 1 μL of solution with the reverse transcriptase enzyme, and 15 μL of RNA. The transcription thermal cycle included a printing phase at 25° C.

Each reverse transcription reaction was performed using 40–60 ng/μL of total RNA, obtained from leaves harvested from different cultivars at different stages of the disease.

### 2.4 High-throughput sequencing

Six libraries of ‘Nocellara del Belice,’ which include three replicates of putative susceptible cultivar (NBT1R1, NBT1R2, and NBT1R3) and three replicates of resistant putative cultivar (NBT3R1, NBT3R2, and NBT3R3) and six libraries of ‘Koroneki,’ which include three replicates of putative susceptible cultivar (KT1R1, KT1R2, and KT1R3) and three replicates of resistant putative cultivar (KT3R1, KT3R2, and KT3R3) were prepared for sequencing. Paired-end sequencing was performed on an Illumina Novaseq 6000 using an S1 Reagent Kit v1.5 (200 cycles, Illumina, Inc.)

### 2.5 Bioinformatics analysis

The RNA-seq reads were quality-checked using fastqc (https://www.bioinformatics.babraham.ac.uk/projects/fastqc/), and the low-quality bases were removed using custom Perl script. Adapter sequences were removed using cutadapt version 2.0 (Martin, 2011). The pre-processed reads were mapped to the *Olea europaea* var. *sylvestris* genome downloaded from NCBI using HISAT2 version 2.1.0 (Kim et al., 2015) and default parameters. The identification of differentially expressed genes was performed using the Cuffdiff algorithm in the Cufflinks version 2.2.1 (Trapnell et al., 2013) pipeline with default parameters. The upregulated and downregulated genes were identified by applying p-value cut-off (<=0.01) and absolute value of log2(FC)>=2. The annotations of the differentially expressed genes were downloaded from uniprot database (https://www.uniprot.org/) and the TAIR ids were obtained through blast searches (blastp) of *Olea europaea* var. *sylvestris* protein sequences against TAIR10 proteins. The blastp result files were parsed using custom Perl script and generated a Mapman (Thimm et al., 2004) mapping file for *Olea europaea* var. *sylvestris* containing the five following categories; (a) Nearly identical: Score ≥ 1000 and e-value = 0, (b) Highly similar: Score ≥ 1000, e-value ≠ 0 OR (Score ≥ 500 and Score < 1000), and e-value = 0, (c) Moderately similar: (Score ≥ 200 and Score < 1000) and e-value! = 0 (d), Weakly similar: (Score ≥ 100 and score < 200), and (e) Very weakly similar: (Score < 100) based on the blastp score and e-value. The metabolic overview and biotic stress gene categories of the differentially expressed genes of each comparison were visualized using the MapMan tool with the generated *Olea europaea* var. *sylvestris Mapman* mapping file. Gene ontology enrichment analysis of the identified differentially expressed genes was performed using the DAVID v6.8 web server (https://david.ncifcrf.gov/). The three classifications of the Gene Ontology (GO) terms (Biological Process, Cellular Component, and Molecular Function) were extracted from the DAVID result using custom Perl script and are given in **Table S3**. GO terms with p-values <=0.05 were considered as significant. The approaches used have already been employed for the analysis of highly complex physiological traits in other species (Benny et al., 2020a; Benny et al., 2022).

### 2.6 Primer design and amplification by RealTime-PCR

Six genes were chosen to confirm the RNA-Seq results and to therefore test gene expression levels using real-time qRT-PCR. Primer pairs were designed with the Primer Premier 3.0 software (https://primer3.ut.ee) (**Table S4**). Reactions were performed in a total volume of 10 μl, containing 0.2 μl of reverse and forward primers, 3.6 μl of ddH_2_O, and 5 μl of the PCR master mix [PCR SuperMix ThermoFisher]. The following PCR thermal cycle was employed: 95°C for 30 s, followed by 40 cycles of 95°C for 15 s, 60°C for 30 s, and a final step of extension of 72° C for 40 s. Melting curve generation (55–95°C) following the final cycle of the PCR was carried out to test the PCR amplification specificity, using real-time PCR cycler Rotor-Gene Q (QIAGEN). The relative expression level of each gene was calculated using the 2^−ΔΔCT^ method using the ‘*housekeeping*’ gene Olest34 (a ribosomal interspacer, GenBank CK087212) (Benitez et al., 2005). All samples were run in triplicates in separate tubes. All data were presented as the mean ± SD after normalization. To perform a comparison of changes in gene relative expressions (GRE) in qRT–PCR tests and RNA-Seq data, Log 2GRE and log 2FC of RNA-Seq were presented.

## 3 Results

### 3.1 RNA sequence data analysis

RNA-seq was used to evaluate transcriptomic changes due to *S. oleagina* infection in the low susceptible olive cultivar Koroneiki and the highly susceptible olive cultivar Nocellara del Belice. Over 23–29 million reads were obtained for the 12 samples, with an average alignment percentage of 83% to the reference *Olea europaea* var. *sylvestris* genome, downloaded from NCBI (**Table S1**). Identification of differentially expressed genes (DEGs) was based on a p-value of <= 0.01, an absolute value of log2(FC)>=2, and fragments per kilobase of exon per million mapped fragments (FPKM) >= 1 in at least one sample. A total of 2,220 DEGs were obtained in all four comparisons (**Table S2**). A total of 475 genes were significantly regulated in the comparison ‘Koroneiki’ T1 vs. ‘Koroneiki’ T3 DEGs, where 341 genes were upregulated and 134 genes were downregulated. A smaller number of DEGs were obtained (229 DEGs) in the comparison ‘Nocellara del Belice’ T1 vs. ‘Nocellara del Belice’ T3 DEGs, where only 39 genes were significantly upregulated and 190 genes downregulated. A total of 678 DEGs (228 upregulated and 450 downregulated) were obtained in the comparison between healthy ‘Nocellara del Belice’ T1 and ‘Koroneiki’ T1. RNA-Seq fastq files were submitted to NCBI SRA under the bioproject PRJNA929711 http://ncbi.nlm.nih.gov/bioproject/PRJNA929711.

### 3.2 RNA-Seq global data analysis and identification of differential gene expression in ‘Koroneiki’ and ‘Nocellara del Belice’

**Figure 1:**
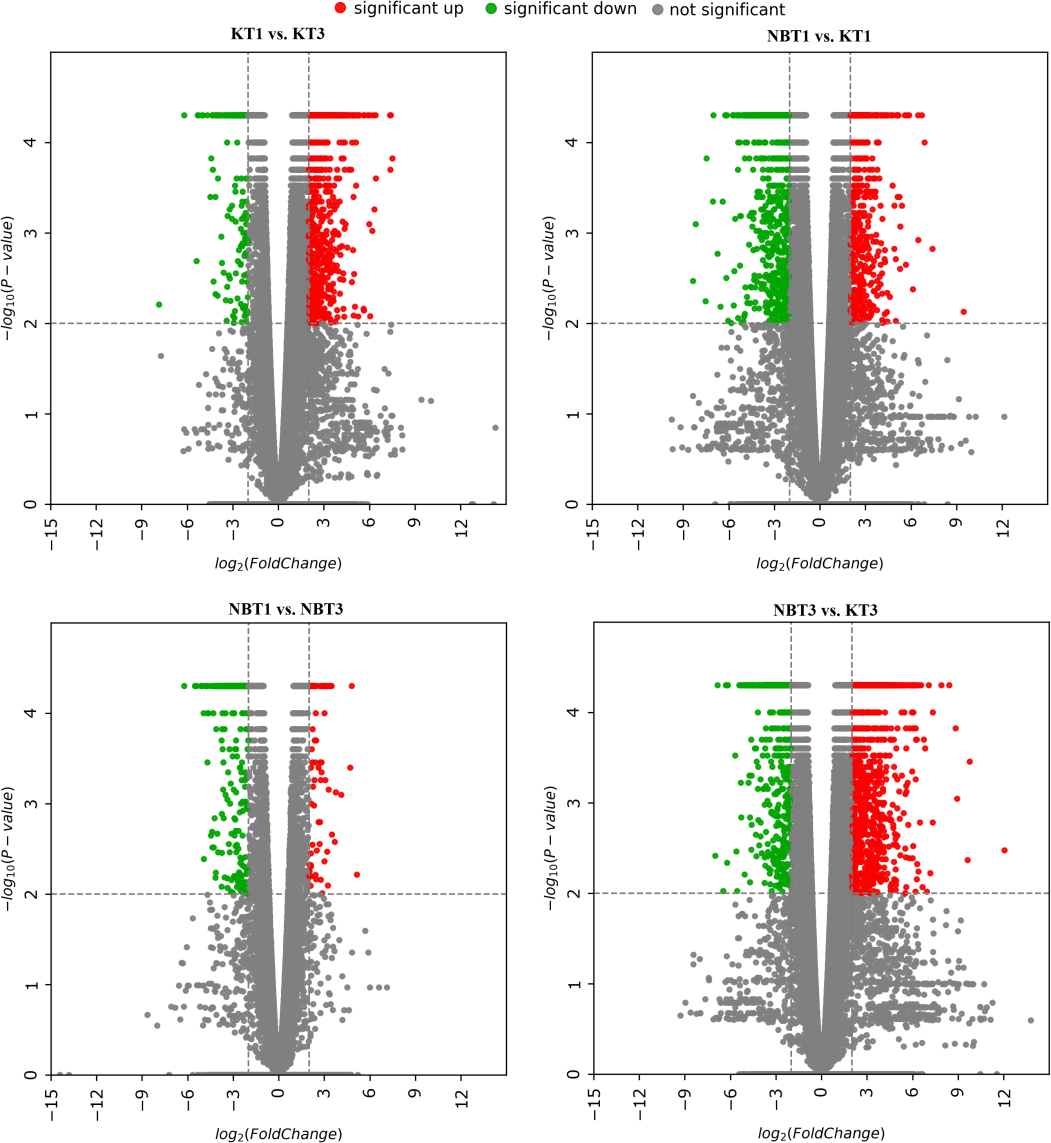
Volcano plots for differentially expressed genes in all four comparisons: KT1 vs. KT3, NBT1 vs. KT1, NBT1 vs. NBT3, and NBT3 vs.KT3. The x-axis shows the log2 fold-change in gene expression between different samples and the y-axis shows the statistical significance of the differences. Upregulated and downregulated genes were filtered (|log 2 (Fold Change)|>= 2, p-value<= 0.01) and are highlighted with red and green dots, respectively; non-significant genes are indicated by grey dots.

A scatter volcano plot was produced to provide an overview of the most interesting DEGs in the four investigated comparisons—KT1 vs. KT3, NBT1 vs. NBT3, NBT1 vs. KT1, and NBT3 vs. KT3. The log2 fold change (FC) was plotted on the x-axis and the negative log10 (p-value) was plotted on the y-axis. The genes with an absolute value of log2(FC)>=2 after applying a p-value cut-off of <=0.01 were considered significant and were highlighted. The grey points show no significant differential gene expression with the absolute value of log2 (FC). The green points show significant downregulated genes, while the red points show significant upregulated genes with the absolute value of log2 (FC) (**Figure 1**).

**Figure 2:**
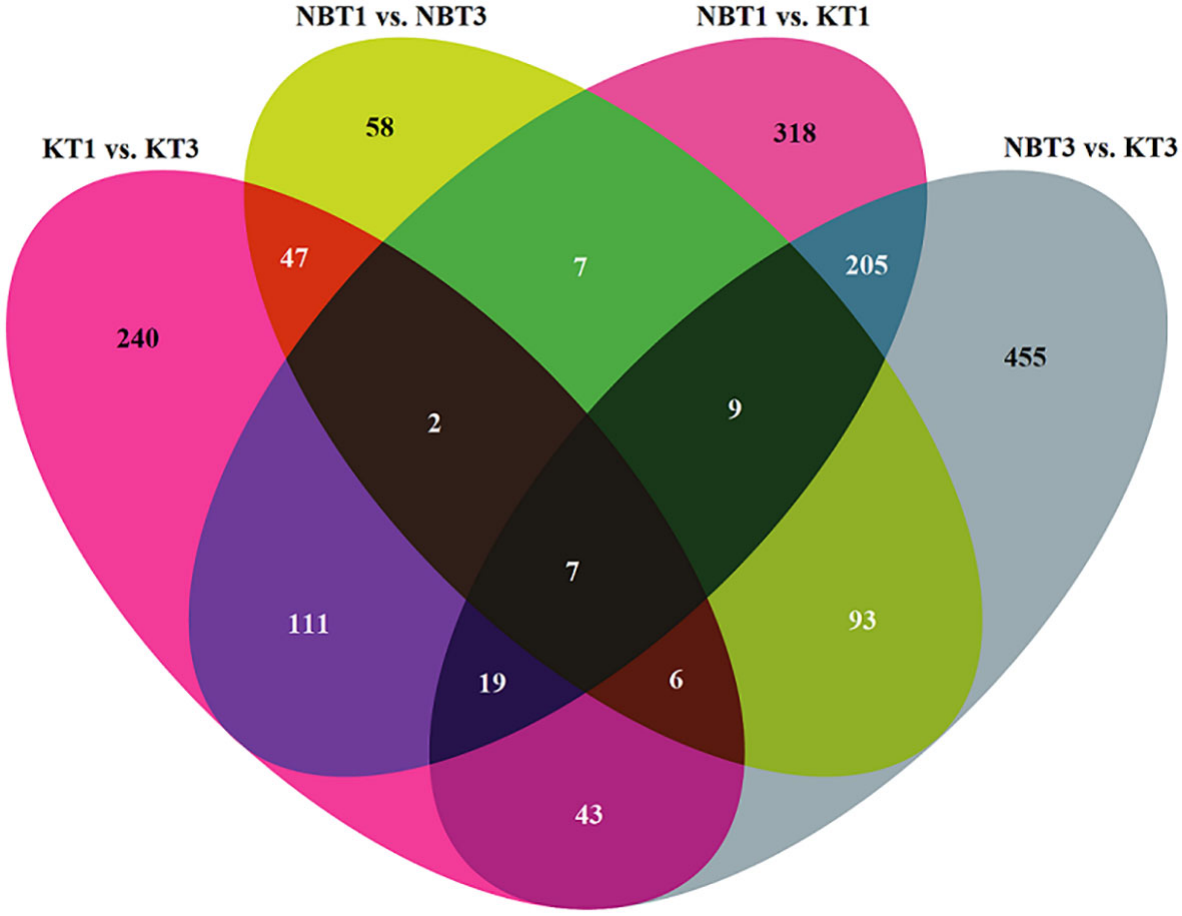
Venn diagram showing the number of specifically and commonly regulated genes in all four comparisons: KT1 vs. KT3, NBT1 vs. KT1, NBT1 vs. NBT3, and NBT3 vs.KT3. The 0 genes were selected by applying the filter: log2(Fold change)>=2 and p-value<=0.01.

Overall, 2,220 differentially expressed genes were found across the four comparisons of interest (KT1 vs. KT3; NBT1 vs. NBT3; NBT1 vs. KT1; and NBT3 vs. KT3), as depicted in a Venn diagram of the comparisons using the cut-off p-value of <=0.01 and absolute log2 Fold change of >=2 (**Table S2**; **Figure 2**).

Gene enrichment analysis was performed for the upregulated and downregulated genes using DAVID (Database for Annotation, Visualization and Integrated Discovery; https://david.ncifcrf.gov/summary.jsp). DAVID enrichment analysis was completed for the comparisons NBT1 vs. KT1, KT1 vs. KT3, and NBT1 vs. NBT3. We applied a p-value cut-off of <=0.05 for the selection of significant DAVID results. The enriched Gene Ontology (GO) terms (Biological process, Cellular component, and Molecular function) obtained from DAVID for the comparison NBT1 vs. KT1 are given in **Figure S2** and those of the remaining comparisons are given in **Figure S3**.

In gene ontology analysis, a greater number of biological processes were downregulated than upregulated in healthy leaves of the cultivar Koroneiki with respect to the cultivar Nocellara del Belice (**Figure S2**, **Table S3**). The transcriptomic studies showed a significant upregulation in “photosynthesis,” “fatty acid metabolic process,” “lipid catabolic process,” and “response to cold,” whereas the “oxidative stress-related processes” and “protein phosphorylation processes” were downregulated. In the cellular component category, the most frequently upregulated GO terms were “chloroplast” and “extracellular region,” and the downregulated GO terms were “integral component of membrane” and “cytoplasm”. Under the molecular function category, “iron ion binding,” “protein domain specific binding,” “phosphatase activity,” and “oxidoreductase activity” were upregulated. The “protein binding,” “ATP binding,” and “protein serine/threonine kinase activities” were downregulated.

### 3.3 ‘Koroneiki’ T1 vs. ‘Koroneiki’ T3 DEGs

**Figure 3:**
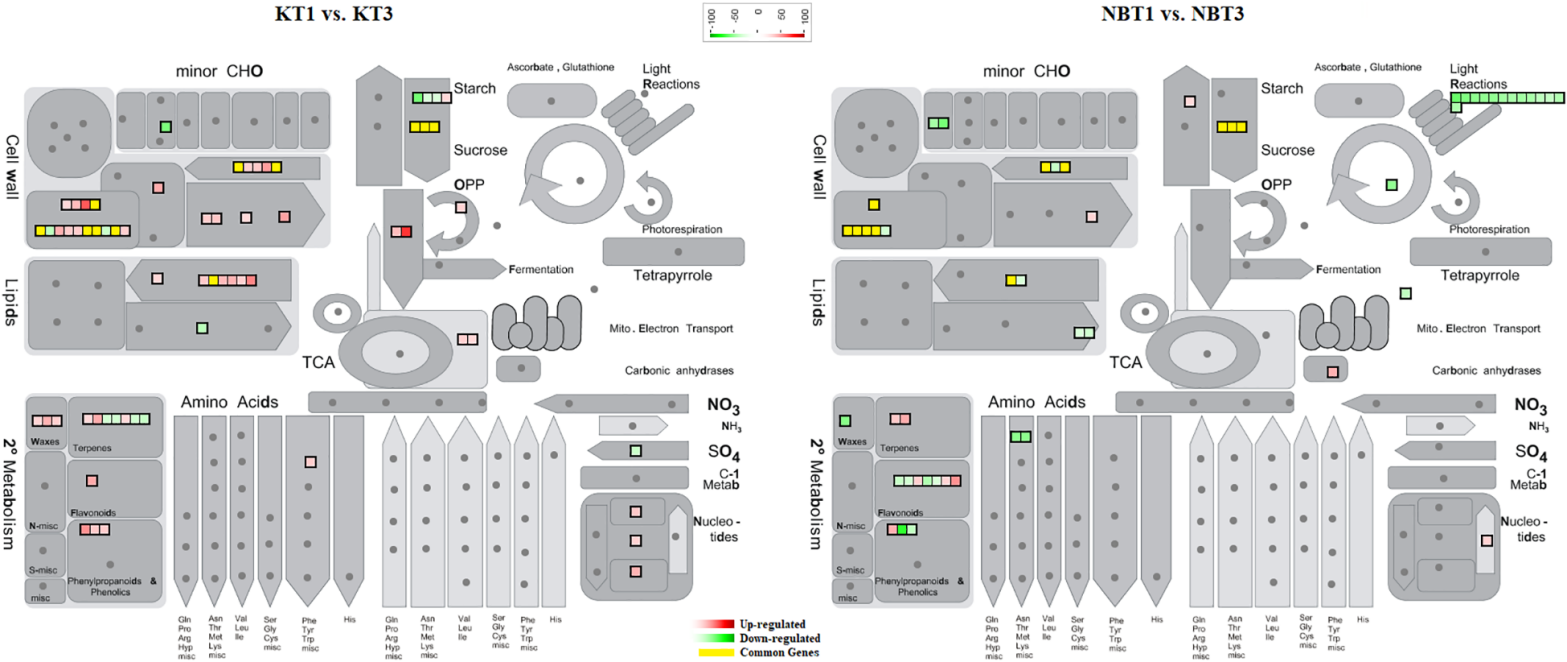
Mapman overview showing differentially regulated genes in ‘Koroneiki’ and ‘Nocellara del Belice’ by *S. oleagina* infection. The upregulated and downregulated genes are highlighted in red and green, respectively, and the genes commonly modulated between ‘Koroneiki’ and ‘Nocellara del Belice’ are highlighted in yellow.

**Figure 4:**
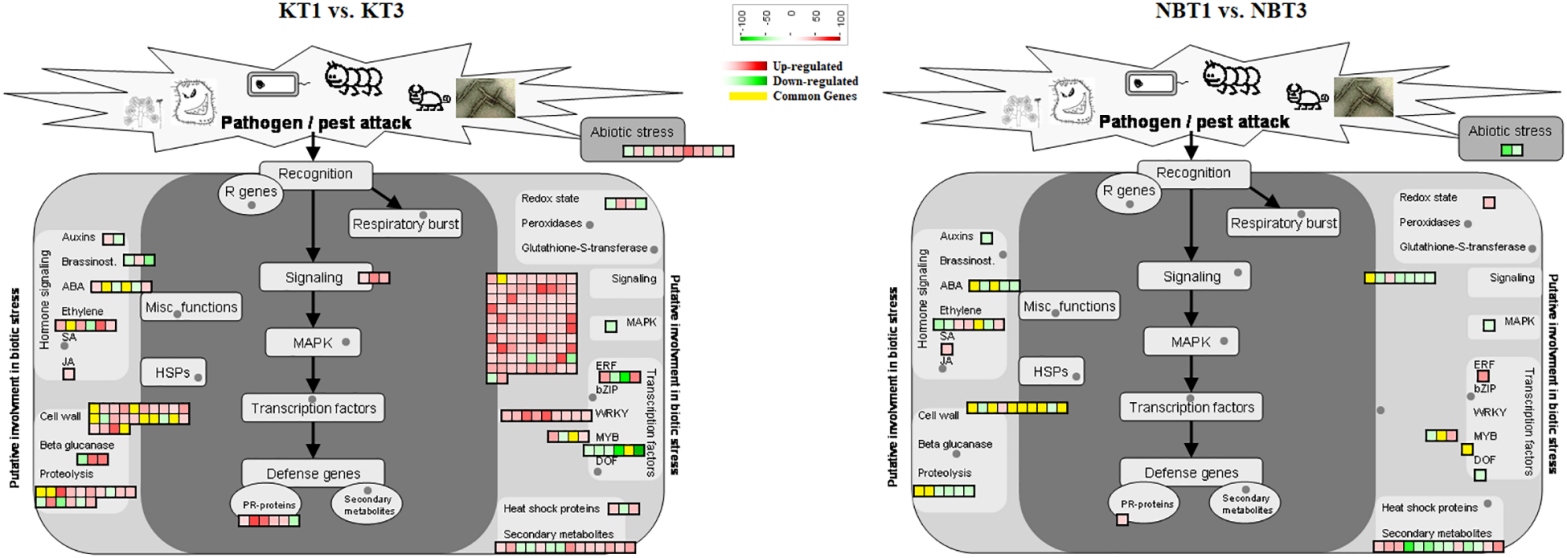
Biotic stress response genes differentially regulated in ‘Koroneiki’ and ‘Nocellara del Belice’ by *S. oleagina* infection. The upregulated and downregulated genes are highlighted in red and green, respectively, and the genes commonly modulated between ‘Koroneiki’ and ‘Nocellara del Belice’ are highlighted in yellow.

**Figure 5:**
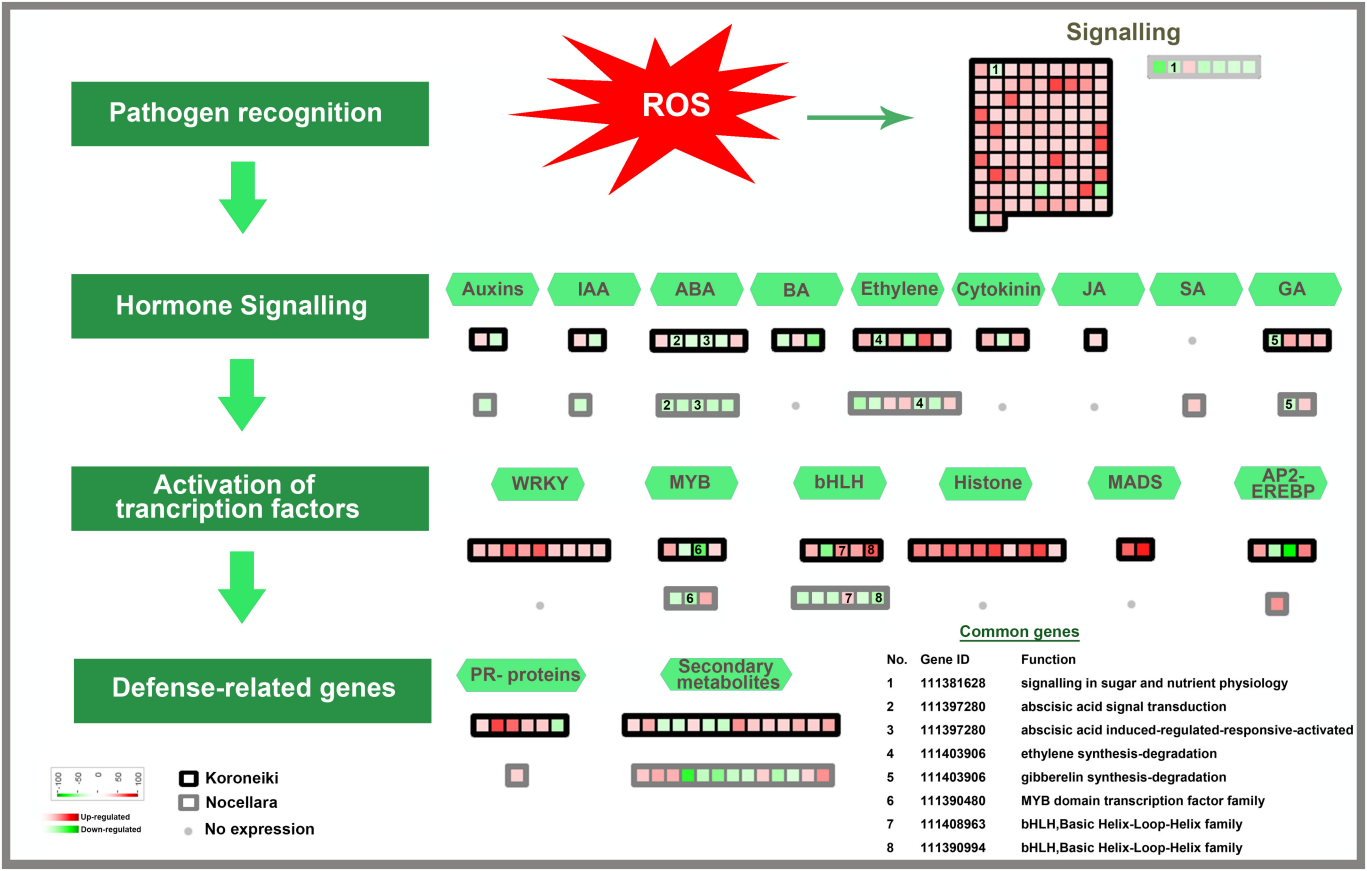
Model for transcription factors regulating biotic stress-signaling pathways. A list of common genes between ‘Koroneiki’ and ‘Nocellara del Belice’ is given. The red gradient shows upregulated genes and the green gradient represents downregulated genes.

In infected leaves, the transcripts expressed included a diverse class of genes mostly related to the classes of “stress signaling and response,” “hormone signaling-related genes,” “chromatin modifications,” “resistance genes,” “transcription factors,” “oxidation-reduction pathways,” “sugar and lipid metabolism,” “ubiquitination genes,” “secondary metabolites,” “polyphenol oxidase (PPO) pathway,” and “cell wall-related pathways”. MapMan software was used to thoroughly visualize the DEGs in different pathways (**Figures 3**–**5**).

Among the class of signaling genes, many upregulated genes are related to abiotic and biotic stress responses such as receptor-like protein kinase (RLKs) genes, including the cysteine-rich receptor-like kinase genes, heavy metal homeostasis and detoxification mechanisms (heavy metal-associated isoprenylated plant protein—HIPP), Calmodulin genes (calmodulin-binding protein 25-like, calmodulin-like protein 8 isoform X2), aquaporins, and TIFY 10b-like genes. It was evident that the upregulation of signaling genes was mediated by the major defense-related phytohormones (Jasmonate (JA), Brassinosteroids, Auxine, ABA, Gibberellins, Cytokinin, and ethylene).

Among the cell-wall sensing genes, extensin genes and wall-associated kinase genes were upregulated.

Among the class of resistance gene analogs or pathogenesis-related (PR) genes, six nsLTPs (non-specific lipid-transfer protein), HD-ZIP genes, seven probable LRR receptor-like serine/threonine-protein kinase genes (ERL2, RBK2, RESISTANT TO DFPM INHIBITION OF ABA SIGNALING 2 (At1g11330), CELLULOSE SYNTHASE LIKE D2 (At1g29720), At3g47570, LYSM RLK1-INTERACTING KINASE 1 (At3g14840), and LRR receptor-like kinase with extracellular Malectin-like Domain 1 (At1g07650)), rust resistance kinase Lr10-like, DOWNY MILDEW RESISTANCE 6-like, glycine-rich protein (GRP), pathogenesis-related leaf protein 6-like, defensin Ec-AMP-D2-like, protein SAR DEFICIENT 1, Thaumatin-like proteins (TLPs)—related to the PR5 family, Ap2-ethylene-responsive transcription factor, GDSL esterase lipase, MADS-box genes, pathogenesis-related proteins (STH-21-like, major allergen Pru ar 1-like), endochitinases, glucan endo-1,3-beta-glucosidases, and many proteinases (metalloendoproteinase 2-MMP-l, aspartic proteinase CDR1-like) were found to be upregulated in infected T3 leaves.

On the contrary, in infected leaves of ‘Koroneiki’ the Plant pathogenesis-related leaf protein 6-like was downregulated.

A large number of genes involved in cell wall biogenesis, remodeling, and cell wall-based defense, including lignin synthesis, were also upregulated: WAT1-related protein (At2g37460), lignans biosynthesis—secoisolariciresinol dehydrogenase-like, lysine-rich arabinogalactan protein 18-like, and CASP-like protein.

Genes involved in ROS and oxidative stress were upregulated (ankyrin repeat-containing protein ITN1-like isoform X2).

Among the phytohormone signaling genes, ethylene-responsive transcription factor TINY-like, salicylic acid-binding protein 2-like, inositol transporter 4, gibberellin-regulated protein 14-like, auxin-induced protein 22D-like, auxin response factor 5-like isoform X4, and Carotenoid cleavage dioxygenases were upregulated.

Abscisic acid receptor PYL4-like and cytokinin dehydrogenase 7-like genes were downregulated.

DEGs involved in protein ubiquitination were also modulated in infected leaves: E3 ubiquitin ligase BIG BROTHER and F-box/LRR-repeat MAX2 were upregulated, whereas E3 ubiquitin-protein ligase RGLG5 was downregulated.

The following transcription factors potentially involved in biotic and abiotic stress responses were up-regulated in infected ‘Koroneiki’ leaves: WRKY6-like, WRKY31, WRKY43, WRKY53, WRKY70, JUNGBRUNNEN 1-like, and MYB family transcription factors (MYB3R-1-like, PHL11, and TF PIF3-like).

The most downregulated transcription factors (TFs) were: RADIALIS-like, GATA transcription factor 21-like, bHLH18-like isoform X2, MYB8-like, ethylene-responsive transcription factor TINY-like, and protein BIC1-like.

The GO analysis (**Figure S3**, **Table S3**) revealed that the terms specific to stress-related processes (biotic and abiotic) were dominant due to *Spilocea oleagina* infection in K1 vs. K3. In cellular components, the membrane-related GO terms “plasma membrane,” “integral component of membrane,” “extracellular region,” “cytosol,” and “cell wall” were significantly upregulated. The molecular function categories “DNA binding” and “protein heterodimerization activity” were highly upregulated due to the fungal infection.

### 3.4 ‘Nocellara del Belice’ T1 vs. ‘Nocellara del Belice’ T3 DEGs

DEGs related to plant immunity, biotic/abiotic stress response, phytoalexin and terpene production, and cell wall-based defense were found to be modulated in healthy (T1) and infected (T3) Nocellara del Belice leaves: exocyst complex component EXO70H1-like, disease resistance response protein 206-like, DMR6-LIKE OXYGENASE 2-like, DOWNY MILDEW RESISTANCE 6-like, serine-type protease inhibitors (proteinase inhibitor PSI-1.2-like), premnaspirodiene oxygenase-like, vetispiradiene synthase 2-like isoform X2, shikimate O-hydroxycinnamoyltransferase related to the phenylpropanoid biosynthesis pathway, bifunctional pinoresinol-lariciresinol reductase-like, shikimate O-hydroxycinnamoyltransferase, and jasmonate O-methyltransferase-like were found to be upregulated in ‘Nocellara del Belice’ T3 (**Figures 3**, **4**). Moreover, two transmitting signals, alpha carbonic anhydrase 1 gene and LRR receptor-like serine/threonine-protein kinase GSO1, and the transcriptional factor MYB8-like TF were also upregulated.

Some signaling genes mediated by the major defense-related phytohormones (Salicylic acid, Gibberellins, ethylene) were also upregulated. Genes related to ABA and Auxin were downregulated.

The downregulated genes in infected leaves included the protein Epidermal Patterning Factor 2-like, GDSL esterase/lipase, chitinase-like protein 1, heavy metal-associated isoprenylated plant protein 24-like, a receptor-like serine/threonine-protein kinase (At5g57670), receptor protein kinase TMK1-like, CBL-interacting protein kinase 2-like, BAHD acyltransferase DCR-like (involved in cuticle formation), cold-regulated 413 plasma membrane protein 2-like, tonoplast dicarboxylate transporter (involved in pH homeostasis), ECERIFERUM 1 gene (involved in wax biosynthesis and alkane biosynthesis), protein NRT1/PTR family 2.11-like, codeine O-demethylase-like, cation/H(+) antiporter 20 isoform X1, plasma membrane ATPase 4-like, asparagine synthetase, carboxylesterase 8, and galactinol synthase 2-like. The transcription factors NAC domain-containing protein 72-like and bHLH149 were downregulated.

In ‘Nocellara del Belice,’ the GO analysis showed that all three functional groups were downregulated (**Figure S2**, **Table S3**) due to *Spilocea oleagina* infection. The major downregulated biological processes were “response to water deprivation,” “response to abscisic acid,” and “response to light stimulus”. In the Cellular component, the terms related to “cytoplasm” and “integral component of membrane” were downregulated.

### 3.5 ‘Koroneiki’ T3 vs. ‘Nocellara del Belice’ T3 DEGs

**Figure 6:**
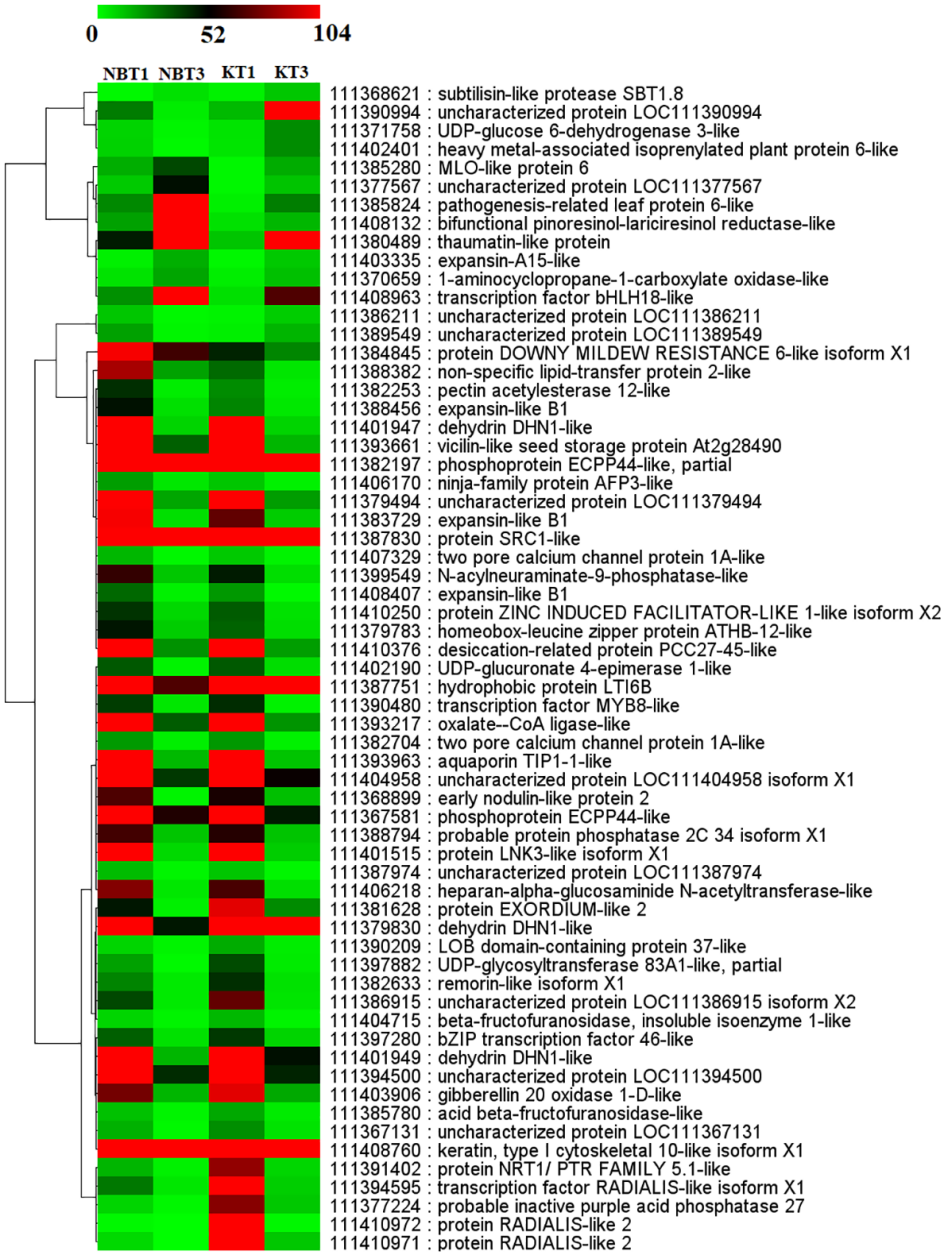
Heatmap showing the expression of 61 common genes present in all four comparisons; KT1 vs. KT3, NBT1 vs. KT1, NBT1 vs. NBT3, and NBT3 vs.KT3. Genes were hierarchically clustered based on a Pearson correlation matrix using FPKM data and average linkage.

Not many genes were found to be common between the infected ‘Koroneiki’ T3 vs. ‘Nocellara del Belice’ T3 (**Figures 3**–**6**). It was noteworthy to discover significant differences between the two cultivars and the uniqueness of genes in both, particularly in ‘Koroneiki’ T3. In infected ‘Koroneiki’ leaves, the upregulation of signaling genes mediated by or in response to the major defense-related phytohormones (JA, Brassinosteroids, Auxine, ABA, Gibberellins, Cytokinin, and Ethylene) was significantly induced, as well as many Pathogenesis-Related (PR) Proteins (non-specific lipid-transfer proteins, HD-ZIP genes, LRR receptor-like serine/threonine-protein kinase genes, and LRR receptor-like kinase with extracellular Malectin-like Domain 1), some of which are usually specifically involved in defense against fungi-rust resistance kinase Lr10-like, glycine-rich protein (GRP), pathogenesis-related leaf protein 6-like, defensin Ec-AMP-D2-like, protein SAR DEFICIENT 1, Thaumatin-like proteins (TLPs), GDSL esterase lipase, pathogenesis-related proteins (STH-21-like, major allergen Pru ar 1-like), Glucan 1,3 Beta Glucosidase, and endochitinases. Genes involved in cell wall modeling, lignine synthesis, and barriers such as WAT1-related protein, lignans biosynthesis-secoisolariciresinol dehydrogenase-like, lysine-rich arabinogalactan protein 18-like, Casparian strip membrane domain (CASP)-like protein can play a fundamental role in conferring biotic and abiotic stress tolerance, indicating the possible role of wall composition on stress resistance. Furthermore, genes involved in the biosynthesis of secondary metabolites, via terpenes and flavonoid biosynthesis (e.g., BAHD acyltransferase gene involved in the production of phenolic secondary metabolites), were upregulated.

In ‘Nocellara del Belice’ T3 infected leaves, few genes involved in biotic/abiotic stress response were upregulated (exocyst complex component EXO70H1-like, disease resistance response protein 206-like, DMR6-LIKE OXYGENASE 2-like, DOWNY MILDEW RESISTANCE 6-like, serine-type protease inhibitors, as well as two transmitting signals; alpha carbonic anhydrase 1 gene and LRR receptor-like serine/threonine-protein kinase GSO1). Furthermore, genes for terpene production were upregulated: premnaspirodiene oxygenase-like, vetispiradiene synthase 2-like isoform X2, shikimate O-hydroxycinnamoyltransferase related to the phenylpropanoid biosynthesis pathway, bifunctional pinoresinol-lariciresinol reductase-like, shikimate O-hydroxycinnamoyltransferase, and jasmonate O-methyltransferase-like.

Moreover, among infected ‘Koroneiki’ T3 and ‘Nocellara del Belice’ T3, the upregulation of TFs showed many differences; for instance, in ‘Koroneiki,’ Ap2-ethylene-responsive transcription factor, MADS-box genes, WRKY6-like, WRKY31, WRKY 43, WRKY 53, WRKY70, JUNGBRUNNEN 1-like, and MYB family transcription factors (MYB3R-1-like, PHL11, and PIF3-like) were upregulated, while in ‘Nocellara del Belice’ only MYB8-like was upregulated, which was downregulated in ‘Koroneiki’.

The common genes are depicted in **Figures 5**, **6**.

### 3.6 Gene validation

To validate the expression, six commonly expressed genes in both the cultivars Nocellara del Belice and Koroneiki were chosen, and Real-Time-PCRs were performed to examine their relative expression levels: LOC111408132 (Bifunctional pinoresinol-lariciresinol reductase-like gene), LOC111384845 (DOWNY MILDEW RESISTANCE 6 gene), LOC111401947 (Dehydrin DHN1-like), LOC111380489 (Thaumatin-like), LOC111410971 (RADIALIS-like 2), and LOC111385280 (MLO-like protein 6). Their expression level agreed with the RNA-seq experiments (**Figure S4**).

## 4 Discussion

When a plant is attacked by pathogens it activates a cascade of genes coding for various receptors, effectors, signaling, and defense molecules. The defense response is localized in cells and tissues, and is characterized by rapid cell death at the pathogen ingress site (Balint‐Kurti, 2019). The production and accumulation of pathogen-related (PR) proteins are part of the innate immune response under biotic and abiotic stress (Finkina et al., 2017). These PR proteins accumulate locally in affected areas and surround even non-infected tissues, providing protection. PR proteins are part of the HR or the systemic acquired resistance (SAR) against different types of infections (Liu et al., 2010). PR proteins, described for the first time in *Nicotiana tabacum* (Van Loon and Van Kammen, 1970) include chitinases, non-specific lipid transfer proteins, β-1,3-glucanases, peroxidases, and thaumatin-like proteins (Van loon and Van strien, 1999; Agrios, 2005; Van Baarlen et al., 2007; Souza et al., 2017; Ali et al., 2018; Vaghela et al., 2022; Wang et al., 2022).

In infected ‘Koroneiki’ leaves, the over-expression of a very high number of PR proteins was evident (**Figures 5**, **6**; **Tables S2**, **S3**), while in ‘Nocellara del Belice’ their expression was more limited.

PR proteins are either extremely acidic or extremely basic and for this reason, they can be highly soluble and reactive (Agrios, 2005). Most PR proteins with an acidic nature are mostly secreted into the extracellular space, whereas PR proteins of a basic nature are mainly located in the vacuole (e.g., Legrand et al., 1987). Some PR proteins have chitinase (Legrand et al., 1987) or β‐1,3‐glucanase activity (e.g., Kauffmann et al., 1987). The enzyme chitinases can hydrolyze chitin, and several are believed to be involved in fungal pathogen defense (Schlumbaum et al., 1986; Kumar et al., 2018). The production of chitinases following fungal infection has often been found in plants, and their function has been validated in transgenic plants in which the expression of cloned chitinase genes has been studied, providing further evidence supporting their involvement in the defense mechanism (Solgi et al., 2015; Kumar et al., 2018; Vaghela et al., 2022). Chitinases hydrolyze chitin/chitosan structural molecules present in various animals and fungi producing small lipo-chito-oligosaccharides (LCOs) (reviewed in Vaghela et al., 2022), which probably function as endogenous stress signal molecules. Interestingly, in infected ‘Koroneiki’ leaves the upregulation of chitinase and β‐1,3‐glucanase genes was manifest. The over-expression of chitinases and β‐1,3‐glucanases in ‘Koroneiki’ T3 therefore suggests the presence of an active antifungal defense system. It can be speculated that the accumulation of chitinases and endoglucanases in infected leaf tissues may interfere with *Spilocea oleagina* growth, presumably weakening the fungal cell wall and reducing the mycelium progression. Glucans or chitin fragments from the cell wall of *Spilocea*, peptides/glycoproteins, or fungal Avr may represent the elicitors inducing PR genes.

The infected ‘Koroneiki’ showed the upregulation of many receptor-like kinases, such as Rust resistance kinase Lr10-like, and many LRR receptor-like serine/threonine-protein kinase genes possibly involved in the signaling recognition of pathogen-derived molecules (PAMP). Rust resistance kinase Lr10-like gene in *Vitis vinifera* is, for example, involved in the recognition of downy mildew (Ricciardi et al., 2022). In addition, a series of signal transduction events, such as lipids and various phytohormones sensing pathogen attack, were also triggered, presumably to activate downstream *Spilocea* immune defense responses. Generally, plants under pathogen attack produce ethylene, Jasmonate (JA), salicylic acid (SA), and reactive oxygen species (ROS) (Yang et al., 1997). Acidic PRs are upregulated by reactive oxygen species and different types of signaling molecules such as salicylic acid (Epple et al., 1995; Reymond and Farmer, 1998; Vaghela et al., 2022), while PRs of a basic nature (defensin, proteinase inhibitors) are upregulated by methyl jasmonate and gaseous phytohormone ethylene (e.g Xu et al., 1994, reviewed in Vaghela et al., 2022). In ‘Koroneiki’ T3, the upregulation of signaling genes mediated by phytohormones (JA, Brassinosteroids, Auxine, ABA, Gibberellins, Cytokinin, and Ethylene) was significantly induced, as well as six non-specific lipid transfer proteins (nsLTPs), considered antimicrobial peptides (AMPs). nsLTPs genes are involved in defense against pathogens, as demonstrated in many studies on transgenic plants. In transgenic wheat, the over-expression of the lipid transfer protein TaLTP5 increased the resistance to the downy mildew *Cochliobolus sativus* and the common root rot *F. graminearum* (Zhu et al., 2012). In transgenic *A. thaliana*, the over-expression of the lipid transfer protein TdLTP4 gene isolated from *T. turgidum* increased the resistance against *Botrytis cinerea* and *Alternaria solani* (Safi et al., 2015). Furthermore, experiments carried out in transgenic carrot plants showed that it is possible to increase the resistance to *Alternaria radicicola* and *B. cinerea* by combining the over-expression of the wheat lipid transfer protein and the barley chitinase 2 gene (Jayaraj and Punja, 2007).

Finkina et al. (2017) proposed a model for the plant defense response mechanism involving nsLTP secretion into the apoplast. Then, nsLTP can bind to other lipid molecules secreted by plants (such as jasmonic acid) or to molecules secreted by pathogens. In this model, when nsLTPs bind to lipid molecules they also interact with LRR serine/threonine protein kinases, as well as with a transmembrane region and a cytoplasmic protein kinase (PK). This interaction causes mitogen-activated protein kinase phosphorylation cascades (MAPK) and possible Ca^2+^-dependent processes, inducing transcription factors and other pathogenesis-related proteins, leading to SAR. This model can fit the molecular scenario observed in ‘Koroneiki’.

Concerning the upregulated TF of cv. Koroneiki (WRKY6-like, WRKY31, WRKY 43, WRKY 53, WRKY70, JUNGBRUNNEN 1-like, MYB family transcription factors (MYB3R-1-like, PHL11, and TF PIF3-like), Ap2-ethylene-responsive transcription factor, and SAR DEFICIENT 1 (SARD1) TF), many have been recognized as key regulators involved in induced systemic resistance (ISR) by simultaneously activating and modulating both the SA and JA/ET signaling pathways (e.g., WRKY70 Jiang et al., 2016; WRKY70 and WRKY53 Hu et al., 2012). Arabidopsis WRKY46, WRKY70, and WRKY53, partially involved in the SA-signaling pathway, positively regulate basal resistance against *P. syringae*, playing synergetic roles in plant basal defense (Hu et al., 2012; Caarls et al., 2015). However, some WRKY TFs (e.g., WRKY6-like, Benny et al., 2020b) and JUNGBRUNNEN 1-like (Thirumalaikumar et al., 2018) have an important role in combined abiotic and biotic stress responses (Bai et al., 2018). SARD1 is a master transcription factor in plant immunity, induced by SA accumulation. Its over-expression is sufficient to activate downstream defense gene expression (Zhang et al., 2010; Huang et al., 2021).

Extensive crosstalk between the hormone signaling pathways allows transcriptional program fine-tuning, leading to resistance to pathogen attacks (Caarls et al., 2015). In ‘Koroneiki,’ the involvement of abscisic acid and ethylene was evident, which may act synergistically with JA-regulated responses, distinctly suppressing SA responses. Auxin, gibberellins, and cytokinins were also upregulated. The hormonal crosstalk in ‘Koroneiki’ resulted in the regulation of defense signaling pathways and the induction of systemic resistance against *Spilocea* through the over-expression of many other important R-genes (GDSL esterase lipase, defensin Ec-AMP-D2-like, pathogenesis-related leaf protein 6-like, Thaumatin-like proteins (TLPs) related to the PR5 family, glycine-rich protein (GRP), MADS-box genes, STH-21-like, major allergen Pru ar 1-like, and many proteinases such as metalloendoproteinase 2-MMP-l and aspartic proteinase CDR1-like). GDSL LIPASE1 (GLIP1) is a defense and ethylene-mediated gene that triggers systemic resistance signaling in plants under fungus and microbial attack; it possesses lipase and antimicrobial activities that directly disrupt fungal spore integrity (Oh et al., 2005).

The AMPs called Plant defensins, found to upregulated in ‘Koroneiki’ T3 leaves, have been purified from several plants and their antifungal activity has been demonstrated (Silva et al., 2014; Aumer et al., 2020; Owens and Doyle, 2021; Struyfs et al., 2021). As a part of the host defense system, it seems that they have a multifaceted mechanism of action, depending on the targeted fungal species (Leannec-Rialland et al., 2022), transferring information between innate and adaptive immune systems (Silva et al., 2014). Some defensins necessitate traversing the cell wall and plasma membrane of the fungi to induce their cell death, others exert their toxic effects without entering the fungal cells. (Leannec-Rialland et al., 2022).

Concerning the pathogenesis-related leaf protein 6-like gene, over-expressed in Koroneiki T3, there is much evidence that this class of proteins is an essential defense component against fungi (reviewed in Hoegen et al., 2002)

In ‘Nocellara del Belice,’ the genes DMR6-LIKE OXYGENASE 2-like, DOWNY MILDEW RESISTANCE 6-like (e.g., Zeilmaker et al., 2015), MLO, and alpha carbonic anhydrase (e.g., Zhou et al., 2020) were upregulated and are recognized as susceptibility factors since it has been reported that they may attenuate or even suppress plant immunity in response to changing environmental factors (Zhou et al., 2020). In *Arabidopsis thaliana*, the inactivation of the DMR6 gene results in increased salicylic acid levels, conferring resistance to diverse pathogens, including bacteria and oomycetes (e.g., Zeilmaker et al., 2015). Nowadays, disruption or inactivation of a single or few susceptible genes is an emergent and faster process to achieve broad-spectrum and durable disease resistance in plants (Acevedo-Garcia et al., 2014; Thomazella et al., 2021). Therefore, in the context of new genome editing technologies (CRISPR/Cas9), these genes may represent possible targets to produce loss-of-function genotypes with improved resistance. TF MYB8, over-expressed in ‘Nocellara del Belice’ infected leaves and downregulated in ‘Koroneiki,’ has been found to be expressed in response to herbivore attacks in other species (Onkokesung et al., 2012).

The Thaumatin-like gene was the unique gene over-expressed in both cultivars. In many species, the over-expression of thaumatin-like protein enhanced resistance to many fungus diseases (Batalia et al., 1996; Benitez et al., 2005; Munis et al., 2010; Acharya et al., 2013; Misra et al., 2016; de Jesús-Pires et al., 2020) as well as to many abiotic stresses (Wang et al., 2022; Su et al., 2021; Sharma et al., 2022). Thaumatin-like proteins possess an acidic cleft that enables them to bind and hydrolyze β-1,3-glucans, conferring antifungal activity (e.g., Wang et al., 2022) or fungal enzyme inhibition (Grenier et al., 1999; Ye et al., 1999).

The RNA-binding protein glycine-rich protein gene, upregulated in ‘Koroneiki’ T3, is known for its anti-fungal and anti-bacterial activity in many species (Czolpinska and Rurek, 2018)

Many proteases, including Metalloendoproteinase genes, were also found to be over-expressed in infected leaves of ‘Koroneiki’; these genes code for proteins involved in the regulation of defense responses against plant pathogen infection (Li et al., 2015). Proteases in other species are reportedly secreted from the cell into the apoplast, a possible site used by pathogens for colonization. According to the latest research by Godson and van der Hoorn, (2021) and Backer et al. (2022), apoplastic proteases can: a) have direct antimicrobial activity; b) activate immune hydrolase; c) be involved in damage-associated molecular pattern release; d) perceive the effectors; e) be involved in the initiation of the HR; and f) be involved in the regulation of both systemic acquired resistance and priming. Furthermore, proteases seem implicated in caspase-like activity in plants, promoting programmed cell death, which is also an important aspect in defense responses (Godson and van der Hoorn, 2021; Backer et al., 2022).

Many genes coding for phenylpropanoid and terpenoid pathways, including those involving lignin, flavonoid, terpene, and phytoalexin production, were found to be over-expressed in ‘Koroneiki’ T3, and to a minor extent in ‘Nocellara del Belice’ T3. Flavonoids, terpenes, and phytoalexins may have antifungal activity and have been shown to play a key role in plant defense against biotic and abiotic stress (Mekuria et al., 2001; Báidez et al., 2007; Rahioui et al., 2009; Aabidine et al., 2010; Giovino et al., 2016; Lanza et al., 2017; Pott et al., 2019; Yadav et al., 2020; Desmedt et al., 2021; Backer et al., 2022). The accumulation of these compounds is more rapid at higher levels in resistant plants, often localized in the infected areas, but is not specific since it may be triggered by many abiotic stresses (Pott et al., 2019; Yousefi et al., 2022).

In addition, many genes encoding for biosynthetic enzymes for cutin, wax, suberin transport, deposition, and remodeling, which are important for epidemical protection against pathogens, were also found to be upregulated in ‘Koroneiki’. Among them, for example, CASPL genes, expressed in the endodermis in a highly tissue-specific manner, encoding for Casparian strip domain proteins (CASPs) induce the formation of the honeycomb-structured lignin brace, which can impede pathogen proliferation (Roppolo et al., 2014; Barbosa et al., 2019).

The Gene Ontology enrichment analysis of all 678 differentially expressed genes in the comparison of ‘Koroneiki’ healthy leaves (T1) with ‘Nocellara del Belice’ T1 revealed that the biological process terms “photosynthesis,” “fatty acid metabolic process,” “lipid catabolic process,” and “response to cold” were significantly enriched (**Figure S2**, **Table S3**). Our study also revealed the enrichment of the GO terms “chloroplast” and “extracellular region,” which demonstrates the plants’ defense response to strengthen the cell wall and increase energy production to support defense-related processes. We also performed GO enrichment analysis on both ‘Koroneiki’ and ‘Nocellara del Belice’ leaves infected by the fungus *Spilocaea oleaginea* (**Figure S3**, **Table S3**). The analysis showed that there were a very high number of GO terms regulated in ‘Koroneiki’ with respect to ‘Nocellara del Belice,’ which shows its strong resistance against the pathogen.

In our work, an overwhelmingly significant contrast in the number of DEGs between ‘Koroneiki’ and ‘Nocellara del Belice’ infected leaves was evident. There is a weak defense response in cv. Nocellara del Belice, indicated by the lack of signaling genes, limited hormonal crosstalk, limited resistance-related gene expression, and the over-expression of putative susceptibility genes. Only a thaumatin-like gene was over-expressed in both cultivars after infection. The thaumatin-like gene is involved in plant defense against both biotic and abiotic stresses (Wang et al., 2022). By using leaves collected at the beginning of March, we determined that the thaumatin-like gene was also employed to detect the early stage of the disease, and it seems a good candidate for this purpose. However, further analysis is needed to confirm this.

In a similar transcriptomic study conducted to explore the resistance mechanism of two avocado rootstocks under the attack of the fungus *Phytophthora cinnamomic*, differing in their susceptibility to the pathogen, a tremendously dissimilar genetic response was described, showing the activation of a plethora of genes in the resilient rootstock and the lack of molecular response in the susceptible rootstock (Backer et al., 2022), consistent with our observation in ‘Koroneiki’ vs. ‘Nocellara del Belice’.

## 5 Conclusion

Many putative defense genes of cv. Koroneiki, such as chitinase, β‐1,3‐glucanase, nsLTPs, GDSL esterase lipase, defensin Ec-AMP-D2-like, STH-21-like, major allergen Pru ar 1-like,Thaumatin-like, metalloendoproteinase, and Casparian strip domain, as well as some TFs (Ap2-ethylene-responsive, SAR DEFICIENT 1, MYB3R-1-like, PHL11, TF PIF3-like, WRKY70, and WRKY53), can be exploited to develop molecular markers for breeding stress-resistant olive genotypes and can become the target of studies involving genetic transformation and genome editing using CRISPR/Cas9 technologies, aiming to acquire resistance/tolerance to *Spilocea oleagina* and possibly other biotic/abiotic stresses. The thaumatin-like gene was uniquely over-expressed in both cultivars, and can be used as a possible marker for early detection of the disease. The genes DMR6-LIKE OXYGENASE 2-like, MLO gene, DOWNY MILDEW RESISTANCE 6-like, and alpha carbonic anhydrase were up regulated in cv. Nocellara del Belice and can be further studied to check whether they represent susceptibility factors and to develop biomarkers for screening the germplasm for vulnerability.

## Data availability statement

The datasets presented in this study can be found in online repositories http://ncbi.nlm.nih.gov/bioproject/PRJNA929711. The names of the repository/repositories and accession number(s) can be found in the article/**Supplementary Material**.

## Author contributions

AM, FM, and AG conceived the study; DT, FB, VI, and AM performed the molecular experiments; FB, AM, and DT analyzed the data; BB performed the bioinformatic analyses; AM took the lead in writing the manuscript; TC, AG, FM, and AM. performed funding acquisition. All authors contributed to the article and approved the submitted version.

## Supplementary material

Supplementary Figure 1
**(A)** Leaves at the T1 stage (no sign of *Spilocea oleagina* disease) of the susceptible cv. Nocellara del Belice (on the left) and the tolerant cv. Koroneiki (on the right) collected on the 24th of September 2021; **(B)** infected leaves at the T3 stage (evident sign of *Spilocea oleagina* disease) collected on the 21st of April 2022.

Supplementary Figure 2 Comparison of the characterization of enrichment of differentially expressed genes in healthy (T1) ‘Koroneiki’ and ‘Nocellara del Belice’. GO enrichment analyses for **(A)** biological processes, **(B)** molecular function, and **(C)** cellular component are shown. The significant GO enrichment terms were selected by applying a p-value cut-off of<=0.05. Each pie segment refers to the percentage of terms present per GO category. Red color=upregulated; green color=downregulated.

Supplementary Figure 3 Characterization of enrichment of differentially expressed genes in ‘Koroneiki’ and ‘Nocellara del Belice’ leaves infected by *Spilocea oleagina* infection (T3). The GO enrichment analyses for **(A)** biological processes, **(B)** molecular function and **(C)** cellular component were shown. The significant GO enrichment terms were selected by applying p-value cut-off <=0.05. Each pie segment refers to the percentage of terms present per GO category. Red color = upregulated; green color = downregulated.

Supplementary Figure 4 qRT-PCR validation of six differentially expressed genes (LOC111408**132** (Bifunctional pinoresinol-lariciresinol reductase-like gene), LOC111384**845** (DOWNY MILDEW RESISTANCE 6 gene), LOC111401**947** (Dehydrin DHN1-like), LOC111380**489** (Thaumatin-like), LOC111410**971** (RADIALIS-like 2), and LOC111385**280** (MLO-like protein 6)). Data from qRT-PCR were normalized relative to the gene Olest34 (a ribosomal interspacer, GenBank CK087212, Benitez et al., 2005). The y-axis denotes the relative fold changes of gene expression calculated from Ct values using the 2^−ΔΔCT^ method; the values are expressed as the means of three replicates ± SD.
