# Supplementary figures and images for: NGS transcriptomic analysis uncovers the possible resistance mechanisms of olive to Spilocea oleagina leaf spot infection
Source: Front Plant Sci. 2023 Jul 17;14:1219580. doi: 10.3389/fpls.2023.1219580 (PMC10388255; doi:10.3389/fpls.2023.1219580)

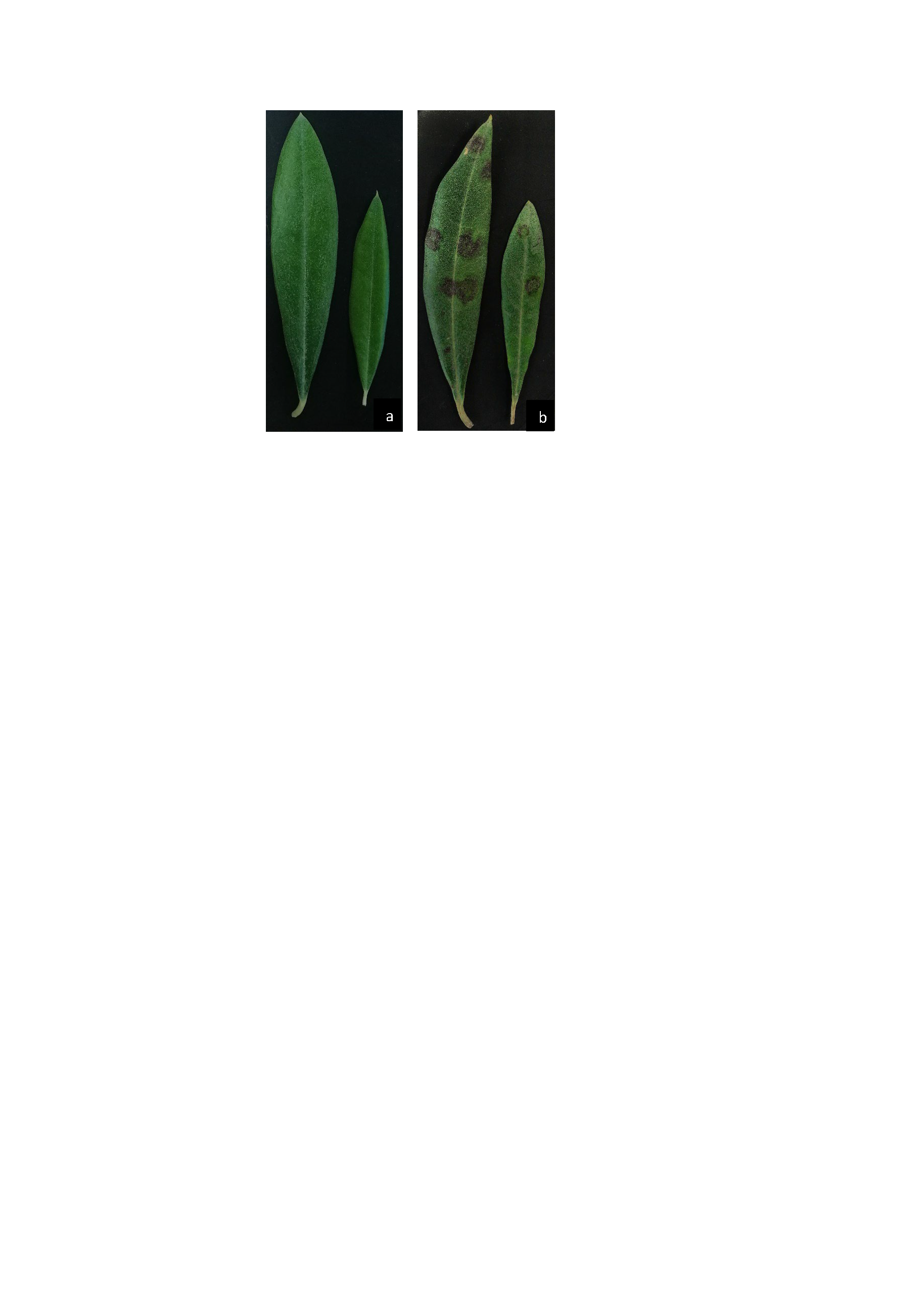

Supplement: Supplementary Figure 1 — (A) Leaves at the T1 stage (no sign of Spilocea oleagina disease) of the susceptible cv. Nocellara del Belice (on the left) and the tolerant cv. Koroneiki (on the right) collected on the 24th of September 2021; (B) infected leaves at the T3 stage (evident sign of Spilocea oleagina disease) collected on the 21st of April 2022. [file Image_1.tiff]

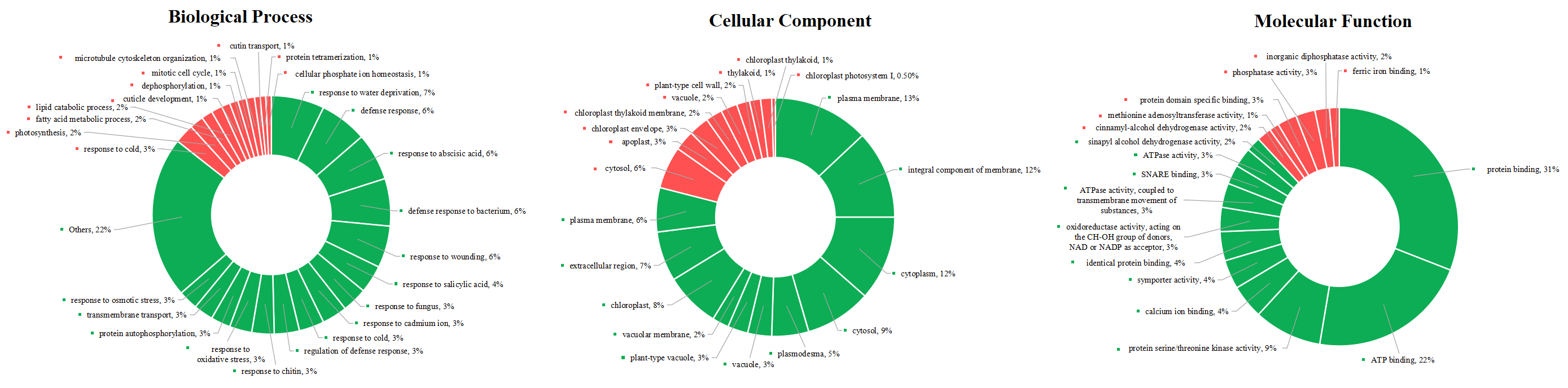

Supplement: Supplementary Figure 2 — Comparison of the characterization of enrichment of differentially expressed genes in healthy (T1) ‘Koroneiki’ and ‘Nocellara del Belice’. GO enrichment analyses for (A) biological processes, (B) molecular function, and (C) cellular component are shown. The significant GO enrichment terms were selected by applying a p-value cut-off of<=0.05. Each pie segment refers to the percentage of terms present per GO category. Red color=upregulated; green color=downregulated. [file Image_2.tif]

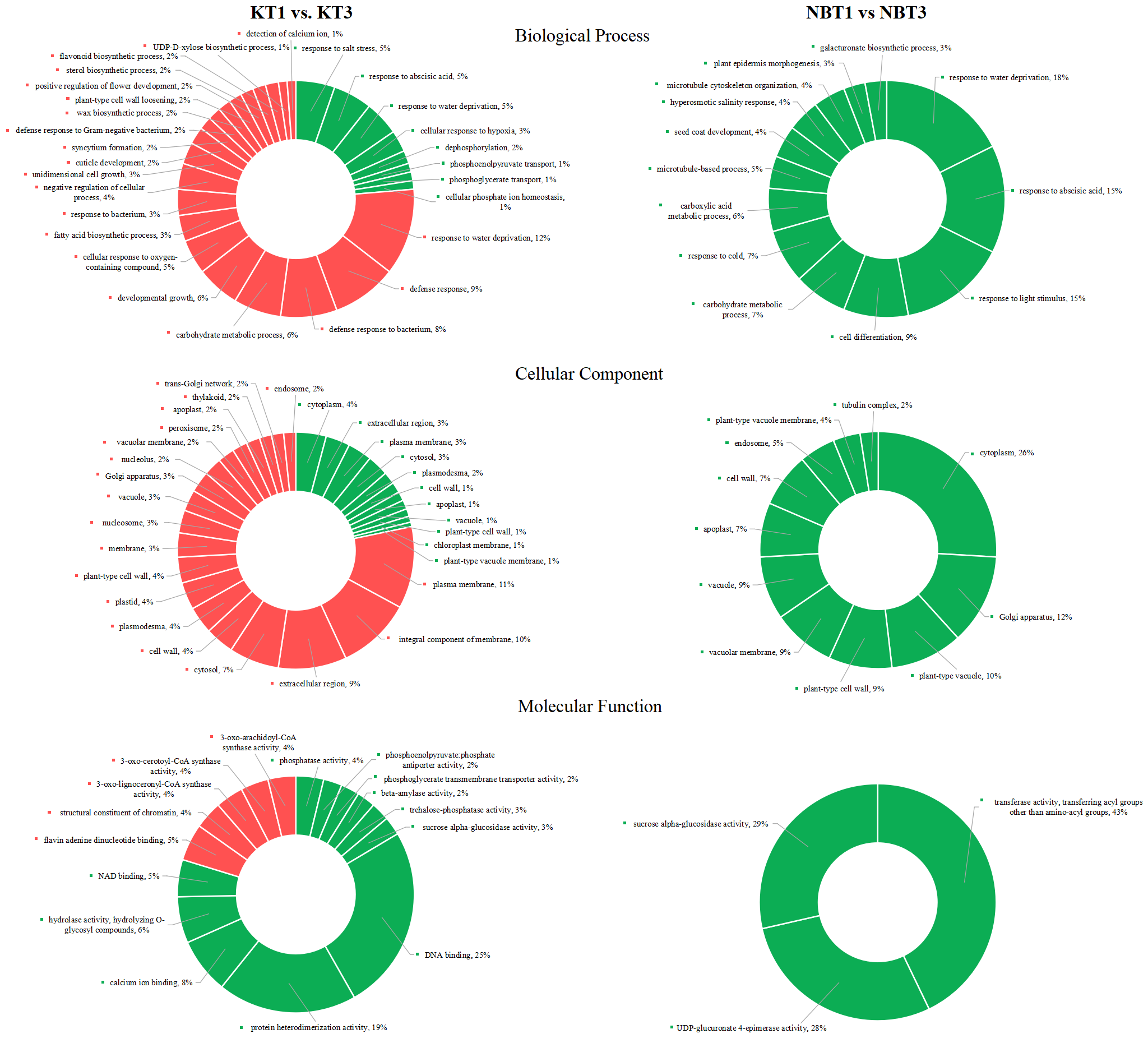

Supplement: Supplementary Figure 3 — Characterization of enrichment of differentially expressed genes in ‘Koroneiki’ and ‘Nocellara del Belice’ leaves infected by Spilocea oleagina infection (T3). The GO enrichment analyses for (A) biological processes, (B) molecular function and (C) cellular component were shown. The significant GO enrichment terms were selected by applying p-value cut-off <=0.05. Each pie segment refers to the percentage of terms present per GO category. Red color = upregulated; green color = downregulated. [file Image_3.tif]

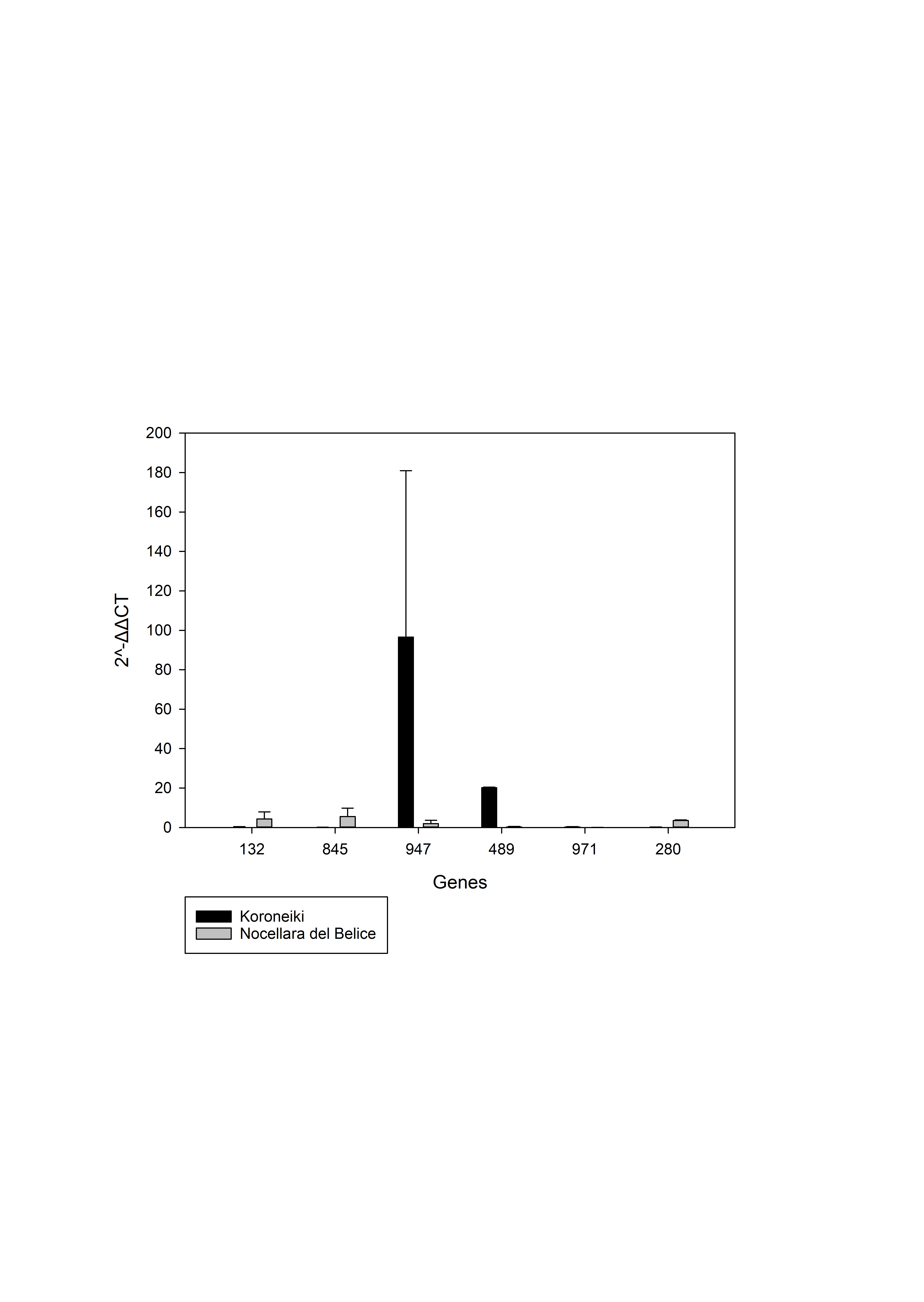

Supplement: Supplementary Figure 4 — qRT-PCR validation of six differentially expressed genes (LOC111408132 (Bifunctional pinoresinol-lariciresinol reductase-like gene), LOC111384845 (DOWNY MILDEW RESISTANCE 6 gene), LOC111401947 (Dehydrin DHN1-like), LOC111380489 (Thaumatin-like), LOC111410971 (RADIALIS-like 2), and LOC111385280 (MLO-like protein 6)). Data from qRT-PCR were normalized relative to the gene Olest34 (a ribosomal interspacer, GenBank CK087212, Benitez et al., 2005). The y-axis denotes the relative fold changes of gene expression calculated from Ct values using the 2−ΔΔCT method; the values are expressed as the means of three replicates ± SD. [file Image_4.jpeg]
